# Tighter bound of quantum randomness certification for independent-devices scenario

**DOI:** 10.1038/s41598-017-15318-4

**Published:** 2017-11-07

**Authors:** Xin-Wei Fei, Zhen-Qiang Yin, Wei Huang, Bing-Jie Xu, Shuang Wang, Wei Chen, Yun-Guang Han, Guang-Can Guo, Zheng-Fu Han

**Affiliations:** 1 Key Laboratory of Quantum Information, CAS, University of Science and Technology of China, Hefei, Anhui 230026 China; 2 Science and Technology on Communication Security Laboratory, Institute of Southwestern Communication, Chengdu, Sichuan 610041 China; 3 State Key Laboratory of Cryptology, P. O. Box 5159, Beijing, 100878 China

## Abstract

Quantum random number generation attracts considerable attention, since its randomness inherently originates in quantum mechanics, but not mathematical assumptions. Randomness certification, e.g. entropy estimation, becomes a key issue in the context of quantum random number generation protocol. We study a self-testing protocol based on dimension witness, with the assumption of independent devices. It addresses the random number extraction problem in a practical prepare-and-measure scenario with uncharacterized devices. However, the lower bound of min-entropy as a function of dimension witness is not tight in existing works. We present a tighter bound of analytic form, by introducing the Lagrangian multiplier method to closely analyze the optimization problem on average guessing probability. Through simulation, it turns out that a significantly higher random number generation rate can be achieved in practice.

## Introduction

Random numbers are widely used in modern science and technology, or even everyone’s daily life. Whether random numbers are of high quality or not depend on what kind of application we use them in. Some applications only require the random sequence to perform well in statistical tests, such as Monte Carlo simulation. Knuth has presented the most commonly used statistical test methods in his famous book “The Art of Computing Programming”, and standard testing suit has been developed by NIST^1^. However, random numbers used in cryptography not only require good statistical properties, but also require security, or unpredictability^2,3^. That is, an attacker who knows part of the random sequence still have no information on other bits, he can only guess with a probability no more than one-half. Both classical cryptography and quantum cryptography require a secure random source^4–6^. A common and convenient way is to generate random sequence by a computer algorithm starting from a seed string, which is reffered to as pseudorandom number generator (PRNG). PRNG cannot be truly random, while security based on algorithm complexity make it not real unpredictable^3^. True random number generator (TRNG) collects unpredictable data from physical process. Specifically, this paper only concerns the quantum random number generation (QRNG)^7^, in which entropy gathering proceeds essentially based on the inherent randomness of quantum mechanics.

Many established methods of quantum optics may be used in QRNG^3,8^, where inherent randomness can be gathered by different quantum parameters of light, such as branching path^9^, time of arrival^10–12^, attenuated pulse^13^, photon counting^14,15^, vacuum fluctuations^16–18^, phase noise^19–21^, and amplified spontaneous emission^22,23^. Randomness certification of these methods may be foiled when the devices are untrusted or far from the theoretical model. It turns out that the device-independent (DI) QRNG^24–28^ offers a solution to the aforementioned problem. By exploiting the quantum violation of Bell inequalities, certified randomness can be achieved without any assumption about the physical implementation. Unfortunately, the observation of a Bell inequality violation without loophole may be extremely challenging, since it requires an unrealistically high detection efficiency to eliminate the detection loophole^28^. Under such a circumstance, compromise solutions termed semi-device-independent QRNG^29,30^ were proposed to explore the tradeoff between loophole-free and implementation. These schemes outperform DI-QRNG by easier implementation and higher performance, with general assumptions such as trusted preparation or measurement devices^31–33^, and a bounded dimension^34–38^.

This paper addresses a semi-device-independent randomness certification problem in the prepare-and-measure scenario. Bowles *et al*.^34^ proposed the so-called dimension witness to bound the quantumness of a prepare-and-measure scenario could behave, with the assumption that the state preparation and measurement devices share no correlations. Based on the aforementioned witness, Lunghi *et al*.^35^ proposed a self-testing QRNG protocol (BQB14 for short)^36^ with a bounded dimension constraint, in which devices had no need to be characterized. The BQB14 derived a lower bound of the min-entropy as a function of dimension witness, and was capable of monitoring the randomness in real time. However, this min-entropy bound was not tight due to the relaxation in derivation procedures, with the domain of dimension witness. As a result, the extracted rate of random bits had a certain gap with the optimal one. We introduce the Lagrangian multiplier method to closely analyze the optimization problem on average guessing probability, and thus a tighter bound of analytic form is presented. As a result, lower guessing probability bound and higher min-entropy can be achieved. We compare the certified randomness between this paper and BQB14 by simulation analysis, it turns out that set-up with the proposed tighter bound achieves a significantly higher certified randomness rate in a practical self-testing QRNG.

## Results

**Figure 1:**
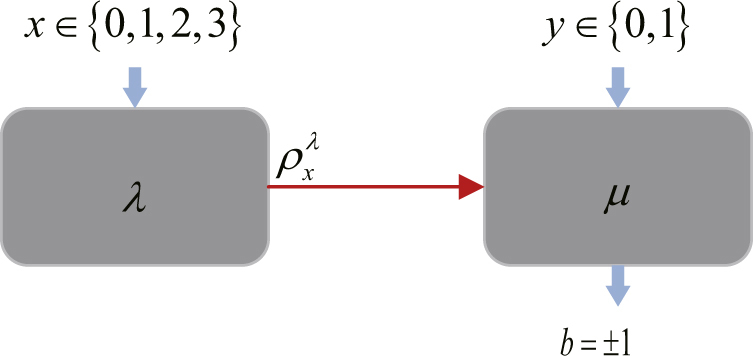
Self-testing QRNG protocol consists of three stages. Data collection: prepare-and-measure experiments are performed with uncharacterized devices, and events {*x*, *y*, *b*} are collected to evaluate the observed probabilities *p*(*b*|*x*, *y*). Entropy monitoring: dimension witness *W* is evaluated by the table of *p*(*b*|*x*, *y*), then the min-entropy can be bounded by an analytic function of variable *W*. Randomness extraction: random numbers are extracted according to the min-entropy in postprocessing.

The prepare-and-measure scenario of QRNG is illustrated in Fig. 1, where a self-testing protocol is performed with uncharacterized devices on both sides. This paper follows the assumptions in BQB14^35^, where imperfection of preparation and measurement devices are modeled by internal random variable *λ* and *μ*. Specifically, it is assumed that devices share no correlations, where *p*(*λ*, *μ*) = *q*_*λ*_ ⋅ *r*_*μ*_ and ∑_*λ*_ *q*_*λ*_ = ∑_*μ*_ *r*_*μ*_ = 1. The random inputs of preparations and measurements are denoted by *x* ∈ {0, 1, 2, 3} and *y* ∈ {0, 1}, and a binary outcome is *b* =±1. In each round of the experiment, a qubit state ${\rho }_{x}^{\lambda }$ is prepared according to random input *x* and internal random variable *λ*, and a similar measurement ${M}_{y}^{\mu }$ is performed then.

In the stage of data collection, events {*x*, *y*, *b*} are collected to evaluate the observed probabilities *p*(*b*|*x*, *y*). Since the observer has no information on the variables *λ* and *μ*, he will observe

1 $$\begin{array}{rcl}p(b|x,y) & = & \sum _{\lambda ,\mu }{q}_{\lambda }{r}_{\mu }p(b|x,y,\lambda ,\mu )\\  & = & {\rm{Tr}}({\rho }_{x}\frac{1+b{M}_{y}}{2})\\  & = & \frac{1}{2}(1+b{\overrightarrow{S}}_{x}\cdot {\overrightarrow{T}}_{y}),\end{array}$$

where

2 $${\rho }_{x}=\sum _{\lambda }{q}_{\lambda }{\rho }_{x}^{\lambda }=\frac{1}{2}(1+{\overrightarrow{S}}_{x}\cdot \overrightarrow{\sigma }),$$

3 $${M}_{y}=\sum _{\mu }{r}_{\mu }{M}_{y}^{\mu }={\overrightarrow{T}}_{y}\cdot \overrightarrow{\sigma }\mathrm{.}$$

The observed states and measurements are denoted by ${\overrightarrow{S}}_{x}$ and ${\overrightarrow{T}}_{y}$ on the Bloch sphere with Pauli vector $\overrightarrow{\sigma }$ = (*σ*_1_, *σ*_2_, *σ*_3_). According to the purification principle of quantum state, ${\overrightarrow{S}}_{x}$ and ${\overrightarrow{T}}_{y}$ can be decomposed on the Bloch sphere by

4 $${\overrightarrow{S}}_{x}=\sum _{\lambda }{q}_{\lambda }{\overrightarrow{S}}_{x}^{\lambda },$$

5 $${\overrightarrow{T}}_{y}=\sum _{\mu }{r}_{\mu }{\overrightarrow{T}}_{y}^{\mu },$$

where ${\overrightarrow{S}}_{x}^{\lambda }$ and ${\overrightarrow{T}}_{y}^{\mu }\,(|{\overrightarrow{S}}_{x}^{\lambda }|=|{\overrightarrow{T}}_{y}^{\mu }|=\mathrm{1)}$ are on the surface of the sphere.

In the stage of entropy monitoring, dimension witness *W* is evaluated by the table of *p*(*b*|*x*, *y*)^34^,

6 $$W=|\begin{array}{cc}p(\mathrm{1|0,0})-p(\mathrm{1|1,0}) & p(\mathrm{1|2,0})-p(\mathrm{1|3,0})\\ p(\mathrm{1|0,1})-p(\mathrm{1|1,1}) & p(\mathrm{1|2,1})-p(\mathrm{1|3,1})\end{array}|.$$

The witness *W* indicates that how quantum is the combination of preparations and measurements, while classical events yield *W* = 0 and quantum events give 0 ≤ |*W*| ≤ 1^34^. To certify the randomness, we derive an upper bound *f* ′ (*W*) of the guessing probability *p* ^*g*^ as an analytic function of *W*, where 0 ≤ *W* ≤ 1. Assuming the choices of preparations and measurements are uniformly distributed, we have the average guessing probability

7 $$\begin{array}{rcl}{p}^{g} & = & \frac{1}{8}\sum _{x,y,\lambda ,\mu }{q}_{\lambda }{r}_{\mu }\mathop{{\rm{\max }}}\limits_{b}p(b|x,y,\lambda ,\mu )\\  &  & \le \,\frac{1}{2}(1+\sqrt{\frac{2-W}{2}})\equiv f^{\prime} (W)\\  &  & \le \,\frac{1}{2}(1+\sqrt{\frac{1+\sqrt{1-{W}^{2}}}{2}})\equiv f(W),\end{array}$$

where *f* ′ (*W*) is tighter than the previous result *f* (*W*)^35^, and the derivation process will be given in next section. Thus, the min-entropy has a tighter lower bound as an analytic function of *W*

8 $${H}_{{\rm{\min }}}=-{\mathrm{log}}_{2}{p}^{g}\ge -{\mathrm{log}}_{2}f^{\prime} (W)\ge -{\mathrm{log}}_{2}\,f(W)\mathrm{.}$$

In the stage of randomness extraction, random numbers are extracted from the raw data. The lower bound $-{\mathrm{log}}_{2}f^{\prime} (W)$ of *H*_min_ is the parameter to determine how many random bits can be extracted in postprocessing.

## Derivation of tighter bound

For given inputs *x*, *y* and local randomness *λ*, *μ*, the guessing probability is given by

9 $$\begin{array}{rcl}{p}_{xy\lambda \mu }^{g} & = & \mathop{{\rm{\max }}}\limits_{b}\,p(b|x,y,\lambda ,\mu )\\  & = & \mathop{{\rm{\max }}}\limits_{b}\,\frac{1}{2}(1+b{\overrightarrow{S}}_{x}^{\lambda }\cdot {\overrightarrow{T}}_{y}^{\mu })\\  & = & \frac{1}{2}(1+|{\overrightarrow{S}}_{x}^{\lambda }\cdot {\overrightarrow{T}}_{y}^{\mu }|)\mathrm{.}\end{array}$$

To certify the randomness, we need to derive an upper bound of the average guessing probability *p* ^*g*^ in (7). Instead of relaxation by inequalities in precious work^35^, we closely maximize the guessing probability with the witness constraint, which is considered to be the reason for the advantage of this paper. Assuming uniformly distributed *x* and *y*, we have

10 $$\begin{array}{rcl}{\rm{\max }}\quad {p}^{g} & = & \frac{1}{8}\sum _{x,y,\lambda ,\mu }{q}_{\lambda }{r}_{\mu }\mathop{{\rm{\max }}}\limits_{b}p(b|x,y,\lambda ,\mu )\\  & = & \frac{1}{2}+\frac{1}{16}\sum _{x,y,\lambda ,\mu }{q}_{\lambda }{r}_{\mu }|{\overrightarrow{S}}_{x}^{\lambda }\cdot {\overrightarrow{T}}_{y}^{\mu }|\\ s\mathrm{.}t\mathrm{.}\quad W & = & |\begin{array}{cc}p(\mathrm{1|0,0})-p(\mathrm{1|1,0}) & p(\mathrm{1|2,0})-p(\mathrm{1|3,0})\\ p(\mathrm{1|0,1})-p(\mathrm{1|1,1}) & p(\mathrm{1|2,1})-p(\mathrm{1|3,1})\end{array}|,\end{array}$$

where *p*(*b* = 1|*x*, *y*) are denoted in (1), (4) and (5).

It is hard to directly derive an analytic solution of the initial problem in (10). Thus, we first focus on a sub-problem of (10) and derive an upper bound on the average guessing probability over the inputs only, where ${p}_{\lambda \mu }^{g}$ is maximized with the witness constraint *W*_*λμ*_:

11 $$\begin{array}{rcl}{\rm{\max }}\quad {p}_{\lambda \mu }^{g} & = & \frac{1}{8}\sum _{x,y}\mathop{{\rm{\max }}}\limits_{b}\,p(b|x,y,\lambda ,\mu )\\  & = & \frac{1}{2}+\frac{1}{16}\sum _{x,y}|{\overrightarrow{S}}_{x}^{\lambda }\cdot {\overrightarrow{T}}_{y}^{\mu }|\\ s\mathrm{.}t\mathrm{.}\quad {W}_{\lambda \mu } & = & ({\overrightarrow{S}}_{01}^{\lambda }\times {\overrightarrow{S}}_{23}^{\lambda })\cdot ({\overrightarrow{T}}_{0}^{\mu }\times {\overrightarrow{T}}_{1}^{\mu }),\end{array}$$

As presented in previous work^34^, we have ${\overrightarrow{S}}_{xx^{\prime} }^{\lambda }=({\overrightarrow{S}}_{x}^{\lambda }-{\overrightarrow{S}}_{x^{\prime} }^{\lambda })/2$. Note that ${\overrightarrow{S}}_{x}^{\lambda }$ must be on the plane spanned by the measurement vectors ${\overrightarrow{T}}_{y}^{\mu }$, so as to maximize the objective function. The angles of ${\overrightarrow{S}}_{x}^{\lambda }$ and ${\overrightarrow{T}}_{y}^{\mu }$ are denoted by {*θ*_0_, *θ*_1_, *θ*_2_, *θ*_3_, *ϕ*_0_, *ϕ*_1_} on this plane. Using the symmetrical nature of the problem, without loss of generality, we set *ϕ*_0_ = 0, *ϕ*_1_ ∈ $[0,\,\frac{\pi }{2}]$, *θ*_0_ ∈ [*ϕ*_0_, *ϕ*_1_], *θ*_1_ = *θ*_0_ + *π*, *θ*_2_ ∈ $[{\theta }_{0},\,{\theta }_{0},\,\frac{\pi }{2}]$, *θ*_3_ = *θ*_2_ + *π*. Thus, problem in (11) can be reduced as:

12 $$\begin{array}{ccl}\mathop{{\rm{\max }}}\limits_{{\theta }_{x},{\varphi }_{y}}{p}_{\lambda \mu }^{g} & = & \frac{1}{2}+\frac{1}{16}\sum _{x,y}|cos({\theta }_{x}-{\varphi }_{y})|\\ \,s\mathrm{.}t\mathrm{.}{W}_{\lambda \mu } & = & \sin ({\theta }_{2}-{\theta }_{0})\cdot \,\sin ({\varphi }_{1}),\end{array}$$

where ${p}_{\lambda \mu }^{g}$ can be simplified as ${p}_{\lambda \mu }^{g}({\theta }_{0},{\theta }_{2},{\varphi }_{1})=\frac{1}{2}+\frac{1}{8}(|\cos ({\theta }_{0})|+|\cos ({\theta }_{0}-{\varphi }_{1})|+|\cos ({\theta }_{2})|+|\cos ({\theta }_{2}-{\varphi }_{1})|)$.

The Lagrange function of problem in (12) is given by

13 $$L({\theta }_{0},{\theta }_{2},{\varphi }_{1},\upsilon )={p}_{\lambda \mu }^{g}({\theta }_{0},{\theta }_{2},{\varphi }_{1})+\upsilon (\sin ({\theta }_{2}-{\theta }_{0})\cdot \,\sin ({\varphi }_{1})-{W}_{\lambda \mu }),$$

where *υ* denotes the Lagrange multiplier. The optimal solution $({\theta }_{0}^{\ast },{\theta }_{2}^{\ast },{\varphi }_{1}^{\ast },{\upsilon }^{\ast })$ should satisfy the gradient equations^39^:

14 $${\nabla }_{{\theta }_{0},{\theta }_{2},{\varphi }_{1},\upsilon }L({\theta }_{0},{\theta }_{2},{\varphi }_{1},\upsilon )=\mathrm{0,}$$

where ${\nabla }_{{\theta }_{0},{\theta }_{2},{\varphi }_{1},\upsilon }L=(\frac{\partial L}{\partial {\theta }_{0}},\frac{\partial L}{\partial {\theta }_{2}},\frac{\partial L}{\partial {\varphi }_{1}},\frac{\partial L}{\partial \upsilon })$. Combining (12) and (14), we get

15 $$\begin{array}{ccc}{p}_{\lambda \mu }^{g} & = & \{\begin{array}{cc}\frac{3+\sqrt{1-{W}_{\lambda \mu }}}{4}, & 0\le {W}_{\lambda \mu }\le \frac{\sqrt{3}}{2}\\ \frac{2+\sqrt{1+{W}_{\lambda \mu }}}{4}, & \frac{\sqrt{3}}{2}\le {W}_{\lambda \mu }\le 1\end{array}\\  & \le  & \frac{1}{2}(1+\sqrt{\frac{2-{W}_{\lambda \mu }}{2}})\\  & \equiv  & f^{\prime} ({W}_{\lambda \mu })\mathrm{.}\end{array}$$

The inequality in (15) holds due to $2\sqrt{1-{W}_{\lambda \mu }}\le 1+\mathrm{(1}-{W}_{\lambda \mu })$ and 0 ≤ *W*_*λμ*_ ≤ 1. The convexity of the witness has been proved in the supplemental material of previous work^35^

16 $$W\le \sum _{\lambda ,\mu }{q}_{\lambda }{r}_{\mu }{W}_{\lambda \mu }\mathrm{.}$$

Thus, the average guessing probability *p* ^*g*^ can be bounded by

17 $$\begin{array}{rcl}{p}^{g} & = & \sum _{\lambda ,\mu }{q}_{\lambda }{r}_{\mu }{p}_{\lambda \mu }^{g}\\  &  & \le \,\sum _{\lambda ,\mu }{q}_{\lambda }{r}_{\mu }f^{\prime} ({W}_{\lambda \mu })\\  &  & \le \,f^{\prime} (\sum _{\lambda ,\mu }{q}_{\lambda }{r}_{\mu }{W}_{\lambda \mu })\\  &  & \le \,f^{\prime} (W)\mathrm{.}\end{array}$$

The inequalities in (17) hold because *f* ′ is concave and decreasing. Finally, we get

18 $$\begin{array}{ll}{p}^{g} & \le \,f^{\prime} (W)=\frac{1}{2}(1+\sqrt{\frac{2-W}{2}})\\  & \le \,f(W)=\frac{1}{2}(1+\sqrt{\frac{1+\sqrt{1-{W}^{2}}}{2}})\mathrm{.}\end{array}$$

To summarize, we first present an analytic solution of the sub-problem in (11), then derive an upper bound of the average guessing probability problem in (10) using the convexity and decrement of the function *f* ′ (*W*). As an analytic function of *W*, the bound *f* ′ (*W*) is tighter than *f* (*W*) in previous work^35^.

## Simulations

In this section, we perform numerical simulations to compare the proposed method and the original one.

**Figure 2:**
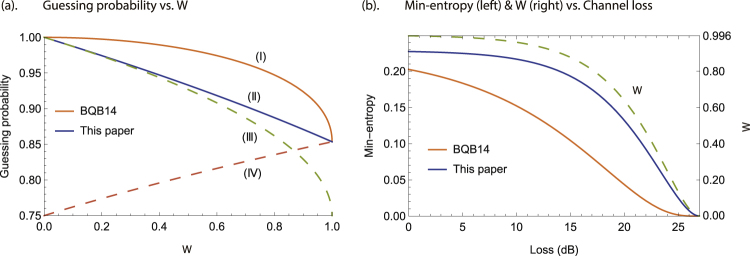
Simulation analysis. (**a**) Comparison of theoretical bounds on average guessing probability. Curve I: upper bound *f* (*W*) in BQB14; Curve II: upper bound *f* ′ (*W*) in this paper; Curve III & IV: intermediate results in (15) as a solution of the sub-problem in (11). (**b**) Comparison of the certified randomness in a practical QRNG with off-the-shelf experimental parameters. Orange line: min-entropy using the bound *f* (*W*) in BQB14; Blue line: min-entropy using the bound *f* ′ (*W*) in this paper. Dashed line: dimension witness *W* corresponding to channel loss is presented on the right axis.

Figure 2(a) gives the comparison of theoretical bounds on average guessing probability. Curve I & II denote the upper bound $f(W)=\frac{1}{2}(1+\sqrt{(1+\sqrt{1-{W}^{2}})\mathrm{/2}})$ in BQB14 and the proposed $f^{\prime} (W)=\frac{1}{2}(1+\sqrt{\mathrm{(2}-W\mathrm{)/2}})$ in this paper, respectively. Curve III & IV indicate the intermediate results $\{\frac{1}{4}(3+\sqrt{1-{W}_{\lambda \mu }}),\,\frac{1}{4}(2+\sqrt{1+{W}_{\lambda \mu }})\}$ in (15) as a solution of the sub-problem in (11). Curve II is derived from Curve III & IV according to the relationship between the initial guessing probability problem in (10) and the sub-problem in (11). As Fig. 2(a) shows, Curve II proposed by this paper is tighter than Curve I in BQB14.

Figure 2(b) presents the comparison of the certified randomness in a practical QRNG with a prepare-and-measure set-up like BB84^40^. Off-the-shelf experimental parameters are set as follows: detection efficiency *η*_*d*_ = 10%, dark count rate *p*_*d*_ = 10 ^−5^, detector misalignment *d*_*e*_ = 1%. Thus the overall QBER *e* = (0.5(1 − 10^− *d*/10^)*p*_*d*_ + *η*_*d*_ *d*_*e*_)/(10^−*d*/10^ + (1 − 10^−*d*/10^)*p*_*d*_). The observed probabilities are assumed as follows: *p*(1|0,0) = 1 − *e*, *p*(1|1,0) = *e*,*p*(1|2,0) = *p*(1|3,0) = 1/2,*p*(1|0,1) = *p*(1|1,1) = 1/2,*p*(1|2,1) = 1 − *e*,*p*(1|3,1) = *e*. In Fig. 2(b), Orange & Blue lines denote the min-entropy using the bound *f* (*W*) in BQB14 and *f*′ (*W*) in this paper, respectively. Note that the dimension witness *W* = 0.996 when loss is zero due to detector misalignment, and the certified randomness has a gap between BQB14 and this paper, even when *W* is close to 1.

## Conclusion

We have presented an analytic bound as a function of dimension witness to estimate the certified randomness, in the prepare-and-measure QRNG with independent devices. Compared with previous works, our work enjoys the advantage of a tighter bound of min-entropy. Simulations have demonstrated that self-testing QRNG with the proposed tighter bound achieves a significantly higher random number generation rate. Benefiting from the better performance of this bound, the self-testing QRNG with similar assumption will accomplish a better balance between security and practicality. There are several issues to be addressed in future. First, the effects of finite-size random number and sampling should be considered. Second, how to guarantee the two-dimensional Hilbert space and independent devices assumptions are essential in practice.
